# Deficiency of Invariant Natural Killer T Cells Does Not Protect Against Obesity but Exacerbates Atherosclerosis in *Ldlr^−/−^* Mice

**DOI:** 10.3390/ijms19020510

**Published:** 2018-02-08

**Authors:** Savitha Subramanian, Leela Goodspeed, Shari Wang, Yilei Ding, Kevin D. O’Brien, Godfrey S. Getz, Alan Chait, Catherine A. Reardon

**Affiliations:** 1 Diabetes Obesity Center for Excellence, Division of Metabolism, Endocrinology and Nutrition, University of Washington, 850 Republican Street Box 35805, Seattle, WA 98109, USA; leelag@uw.edu (L.G.); sawang@uw.edu (S.W.); yilei@uw.edu (Y.D.); achait@u.washington.edu (A.C.); 2 Division of Cardiology, University of Washington, Seattle, WA 98195, USA; cardiac@uw.edu; 3 Department of Pathology, University of Chicago, Chicago, IL 60637, USA; getz@bsd.uchicago.edu (G.S.G.); reardon@uchicago.edu (C.A.R.)

**Keywords:** natural killer T cells, adipose tissue, atherosclerosis, inflammation

## Abstract

Obesity is a chronic inflammatory state characterized by altered levels of adipose tissue immune cell populations. Natural killer T (NKT) cells are CD1d restricted lymphocyte subsets that recognize lipid antigens whose level decreases in obese adipose tissue. However, studies in mice with deficiency or increased levels of NKT cells have yielded contradictory results, so the exact role of these cells in obesity and adipose tissue inflammation is not yet established. We previously showed that *Ldlr^−/−^* mice with excess invariant NKT (iNKT) cells demonstrate significant weight gain, adiposity, metabolic abnormalities, and atherosclerosis. The current study evaluates the effects of NKT cell deficiency on obesity, associated metabolic changes, and atherosclerosis in *Jα18^−/−^Ldlr^−/−^* (lacking iNKT cells) and *Cd1d^−/−^Ldlr^−/−^* (lacking invariant and type II NKT cells) mice, and control mice were fed an obesogenic diet (high fat, sucrose, cholesterol) for 16 weeks. Contrary to expectations, *Ja18^−/−^Ldlr^−/−^* mice gained significantly more weight than *Ldlr^−/−^* or *Cd1d^−/−^Ldlr^−/−^* mice, developed hypertriglyceridemia, and had worsened adipose tissue inflammation. All the mice developed insulin resistance and hepatic triglyceride accumulation. *Ja18^−/−^Ldlr^−/−^* mice also had increased atherosclerotic lesion area. Our findings suggest that iNKT cells exacerbates the metabolic, inflammatory, and atherosclerotic features of diet-induced obesity. Further work is required to unravel the paradox of an apparently similar effect of iNKT cell surplus and depletion on obesity.

## 1. Introduction

Obesity is now well characterized as a chronic inflammatory state with a variety of cellular changes occurring at the level of adipose tissue [1,2,3,4]. Adipose tissue is a dynamic endocrine organ with a distinct collection of immune cells in its stroma that play an important role in maintaining homeostasis. As obesity develops, the levels and function of immune cell types in adipose tissue is altered. This includes increased levels of M1 macrophages, Th1 cells, CD8+ T cells, natural killer cells, and neutrophils, and decreased levels of natural killer T (NKT) cells, M2 macrophages, T and B regulatory cells, and type 2 innate lymphoid (ILC2)cells. The resultant differential cellular activation and milieu of cytokines and other factors are thought to propagate the metabolic dysregulation [5,6].

NKT cells are a specialized subset of T lymphocytes that recognize glycolipid antigens presented by the major histocompatibility complex (MHC) class I-like molecule CD1d [7,8,9,10]. The most abundant NKT cells are designated type I or invariant NKT cells (iNKT). They express semi-invariant T cell receptor α-(TCRα) chains coupled with a limited repertoire of TCRβ chains. In mice, the TCRα on iNKT cells is Vα14-Jα18. A less abundant population of NKT cells are designed type II NKT cells. They express more diverse TCRs, and hence no specific surface marker for these cells currently exists; these cells are designated type II NKT cells. Both cell types are CD1d restricted, and recognize lipid antigens loaded onto CD1d on the surface of antigen presenting cells (macrophages and dendritic cells). CD1d is also expressed on the surface of epithelial cells, such as adipocytes, which are also thought to activate iNKT cells [11,12]. The differences in surface T cell receptors can be employed to distinguish effects of iNKT cells from type II NKT cells. Thus, while mice lacking the CD1d molecule are deficient in both types of NKT cells, mice lacking the Jα18 chain of the T cell receptor are selectively deficient in iNKT cells.

The precise nature of lipid antigens recognized by NKT cells remains unclear, and is an area of intense investigation [13,14]. The first lipid identified as an antigen for iNKT cells was α-galactosylceramide (αGalCer), which remains the most potent activator of iNKT cells. Several microbial lipid ligands have been recently identified; however, the search for physiologically relevant lipids from pathogens or endogenous sources recognized by iNKT cells continues. The ligands for type II NKT cells are thus far even less well understood [15].

In both mice and humans, visceral adipose tissue is enriched in iNKT cells where they represent about 20% of total T cells [16]. Several groups, including ours, have demonstrated that iNKT cell numbers decrease in adipose tissue as obesity develops in mice fed a high fat diet [11,16,17,18,19]. Similar changes are also observed in adipose tissue from obese subjects [11,16,18]. iNKT cells are found in many tissues, and are heterogeneous in their phenotypes. Adipose tissue iNKT cells are distinguished from those in other tissues, such as the liver and spleen, in that they do not express the transcription factor promyelocytic leukemia zinc finger (PLZF) [20]. By contrast, they express the transcription factor E4BP4, which drives the expression of IL-10. The iNKT cells of the adipose tissue (NKT10) have some of the characteristics of regulatory cells, though they do not express Foxp3 [21].

To study the contribution of NKT cells to obesity, several groups have utilized the available models of NKT cell deficiency, namely *Ja18^−/−^* and *Cd1d^−/−^* mice [11,16,19,22,23,24,25,26,27]. These deficient models, when fed a high fat diet generally, though not in all reports, develop more profound obesity and associated metabolic changes than their wild type controls [28]. The rescue of the obesity and metabolic phenotype by the adoptive transfer of purified hepatic iNKT cells into obese mice supports the importance of iNKT cells in protecting against the obesity induced metabolic phenotype [16]. Discrepancies in results that have been observed among various research groups are likely attributable to differences in the age of animals, diets utilized, and environmental factors. Differences in the phenotype of *Ja18^−/−^* and *Cd1d^−/−^* mice could represent a measure of the effects of type II NKT cells. 

In most studies, obesity is induced with a high fat diet without supplementary dietary cholesterol, and in animals expressing the low density lipoprotein receptor (LDLR), a cell surface protein responsible for the high affinity uptake of plasma LDL. The feeding of an obesogenic diet (high fat, high sucrose) supplemented with additional cholesterol (HFHSC) to mice in the LDLR deficient (*Ldlr^−/−^*) background results in much more profound hyperlipidemia than in wild type mice, and enables the simultaneous evaluation of obesity and atherosclerosis [29]. We recently reported that the presence of an increased complement of iNKT cells in Vα14 transgenic mice on an *Ldlr^−/−^* background (Vα14tg *Ldlr^−/−^*) that were fed the HFHSC diet did not protect against diet-induced obesity, and in fact resulted in increased weight gain, insulin resistance, and aortic atherosclerosis [17]. In the current study, we fed this diet to *Ldlr^−/−^* mice lacking only iNKT cells (*Ja18^−/−^Ldlr^−/−^)* or lacking both NKT cells subsets (*Cd1d^−/−^Ldlr^−/−^*), and show that both, but especially the former, are more obese than the control *Ldlr^−/−^*mice. The *Ja18^−/−^Ldlr^−/−^* mice also exhibit more atherosclerosis than control mice. Thus, iNKT cell deficient mice are also not protected against diet-induced obesity or atherosclerosis.

## 2. Results

### 2.1. Natural Killer T (NKT) Cell Deficiency Is Associated with Weight Gain in Ldlr^−/−^ Mice on a High Fat, High Sucrose Cholesterol Enriched (HFHSC) Diet

**Figure 1:**
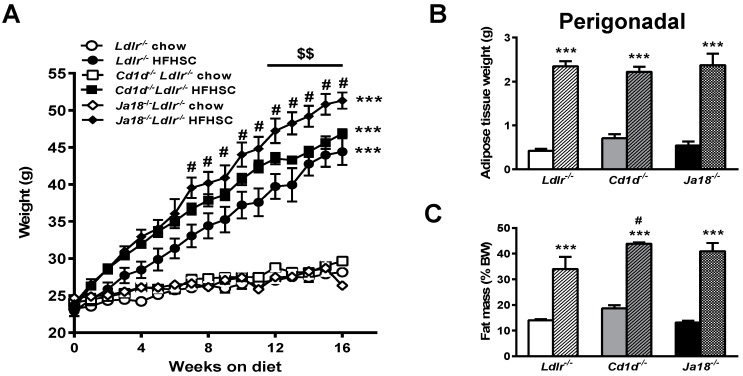
Invariant natural killer T (iNKT) cell deficient *Ja18^−/−^Ldlr^−/−^* mice exhibit increased weight gain. (**A**) Significantly increased weight gain in *Ja18^−/−^Ldlr^−/−^* mice fed high fat, high sucrose cholesterol enriched (HFHSC) diet for 16 weeks; (**B**) Perigonadal adipose tissue weights; (**C**) Body composition analysis revealed increased generalized fat distribution in all groups of obese mice. Data represent means ± standard error of the mean (SEM), *n* = 10 mice per group. *** *p* < 0.001 vs. lean mice of corresponding group, # *p* < 0.01 vs. HFHSC-fed *Ldlr^−/−^* mice, $$ *p* < 0.001 vs. HFHSC-fed *Cd1d^−/−^Ldlr^−/−^* mice. Open symbols and solid bars—chow-fed animals; closed symbols and hatched bars—HFHSC-fed animals. BW: body weight.

We have previously shown that iNKT cell numbers are reduced in *Ldlr^−/−^* mice fed a high fat, high sucrose cholesterol enriched (HFHSC) diet [17]. To evaluate the effects of NKT cell deficiency in obesity associated metabolic derangements, we utilized a loss of function approach using iNKT cell deficient *Ja18^−/−^Ldlr^−/−^* and both iNKT cell and type II NKT cell deficient *Cd1d^−/−^Ldlr^−/−^* mice. The mice were placed on standard chow or HFHSC diet for 16 weeks. *Ldlr^−/−^* mice fed a HFHSC diet develop obesity, hyperinsulinemia, hyperlipidemia, and significant atherosclerosis [29]. No differences in body weight or adiposity were observed among chow-fed animals of any group (Figure 1A); however, when challenged with the HFHSC diet, weight gain was significantly higher in *Ja18^−/−^Ldlr^−/−^* mice compared to *Cd1d^−/−^Ldlr^−/−^* as well as control *Ldlr^−/−^* mice (*p* < 0.001, Figure 1A). Food intake was equivalent between the HFHSC diet groups. There were no significant differences in the perigonadal (intra-abdominal) fat pad weights between the obese NKT cell deficient mice and *Ldlr^−/−^* control mice (Figure 1B), suggesting an increase in other fat depots. While body composition analysis revealed increased generalized body fat distribution in all groups fed the HFHSC diet (Figure 1C), the *Cd1d^−/−^Ldlr^−/−^* mice had relatively more body fat than did the *Ldlr^−/−^* mice and the proportion of fat mass in the *Ja18^−/−^Ldlr^−/−^* mice trended in the same direction (*p* = 0.1). No differences in lean body mass were observed between the obese groups (S. Subramanian, University of Washington, Seattle, WA, USA, 2015) (not shown). Thus, the absence of NKT cells does not prevent weight gain in *Ldlr^−/−^* mice fed the HFHSC diet.

### 2.2. Obesity-Induced Metabolic Abnormalities Are Observed in the Presence and Absence of NKT Cells

**Figure 2:**
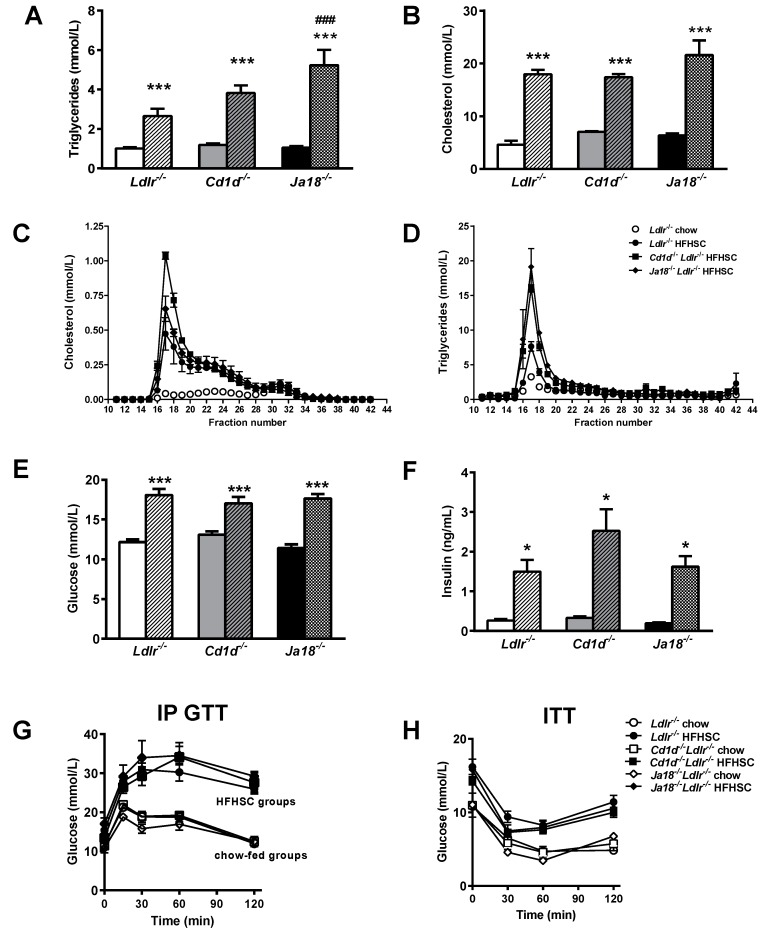
Effects of NKT cell deficiency on metabolic abnormalities that accompany obesity. (**A**) Plasma triglyceride and (**B**) cholesterol levels in mice fed chow or HFHSC diets. Plasma lipoprotein cholesterol (**C**) and triglyceride (**D**) distribution in lean *Ldlr*^−/−^ and HFHSC-fed *Ja18^−/−^Ldlr^−/−^, Cd1d^−/−^Ldlr^−/−^,* and *Ldlr^−/−^* mice. (**E**) Plasma fasting glucose, (**F**) fasting insulin, and (**G**) intraperitoneal (ip) glucose tolerance test. (**H**) Insulin tolerance test. *n* = 10 per group. * *p* < 0.05 vs. corresponding lean mice, *** *p* < 0.001 vs. corresponding lean mice, ### *p* < 0.0001 vs. HFHSC-fed *Ldlr^−/−^* mice. Open symbols and solid bars—chow-fed animals; closed symbols and hatched bars—HFHSC-fed animals.

Hypercholesterolemia and hypertriglyceridemia developed in all groups of mice on the HFHSC diet. However, hypertriglyceridemia was amplified in *Ja18^−/−^Ldlr^−/−^* mice, while plasma cholesterol levels were equivalently elevated in all three groups of obese mice (Figure 2A,B). Lipoprotein profiles showed increased VLDL/IDL particles in the HFHSC-fed *Ja18^−/−^Ldlr^−/−^* and *Cd1d^−/−^Ldlr^−/−^* mice (Figure 2C,D). Fasting hyperglycemia and hyperinsulinemia was observed in all three groups of mice on HFHSC feeding (Figure 2E,F). No differences were observed on glucose or insulin tolerance testing (Figure 2G,H). No differences in lipids or insulin sensitivity were observed in the lean chow-fed animals across the three groups. Thus, the metabolic abnormalities associated with obesity are not improved by the absence of NKT cells. 

### 2.3. Adipose Tissue Inflammation Is Worsened in Obese Ja18^−/−^Ldlr^−/−^ Mice Devoid of iNKT Cells

**Figure 3:**
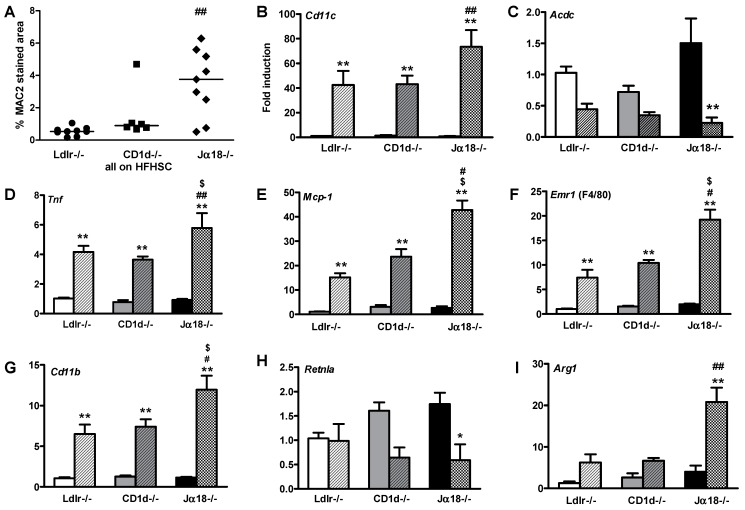
Adipose tissue inflammation is enhanced in obese *Ja18^−/−^Ldlr^−/−^* mice. (**A**) Quantification of macrophage Mac2 staining of adipose tissue; (**B**–**I**) Expression level of genes in whole perigonadal adipose tissue expressed relative to lean chow-fed *Ldlr^−/−^* mice. *n* = 5–10 per group. * *p* < 0.05 vs. corresponding lean group, ** *p* < 0.01 vs. corresponding lean group, # *p* < 0.05 vs. HFHSC-fed *Ldlr^−/−^* mice, ## *p* < 0.01 vs. HFHSC-fed *Ldlr^−/−^*, $ *p* < 0.05 vs. HFHSC-fed *Cd1d^−/−^Ldlr^−/−^* mice. Solid bars—chow-fed animals; closed symbols and hatched bars—HFHSC-fed animals.

Since activated iNKT cells interact with tissue macrophages [28], we examined whether the absence of iNKT cells influences the macrophage content and inflammatory state of adipose tissue. Obese *Ja18^−/−^Ldlr^−/−^* mice accumulated more macrophages in perigonadal (intra-abdominal) adipose tissue compared to controls, as demonstrated by increased immunostaining for the macrophage specific protein Mac2 (*p* < 0.01, Figure 3A). In addition, the expression of *Cd11c*, which is found on macrophages and other immune cells, such as dendritic cells and natural killer cells, is also increased (Figure 3B). Analysis of gene expression in whole adipose tissue revealed that obese *Ja18^−/−^Ldlr^−/−^* mice had significantly reduced expression of adiponectin and increased *Tnfa,* compared to their lean counterparts (Figure 3C,D). The obese *Ja18^−/−^Ldlr^−/−^* mice also showed increased expression of the monocyte chemotactic factor 1 (*Mcp1*) gene (Figure 3E) and macrophage specific genes, such as *Emr1* (F4/80) and *Cd11b* (Figure 3F,G), when compared to control *Ldlr^−/−^* and *Cd1d^−/−^Ldlr^−/−^* mice. Overall, the accumulated macrophages showed a phenotype of pro-inflammatory M1 activation, as evidenced by increased *Tnfα* expression with decreased expression of *Retnla* (Figure 3H)*,* an anti-inflammatory activation M2 marker. However, *Arg1*, a M2 activation marker, is increased in the adipose tissue from the obese *Ja18^−/−^Ldlr^−/−^* mice (Figure 3I). Taken together, these findings indicate an increase in adipose inflammatory changes in obese *Ja18^−/−^Ldlr^−/−^* mice lacking iNKT cells.

### 2.4. Hepatic Steatosis Is Observed in Obese Mice in the Presence and Absence of iNKT Cells

**Figure 4:**
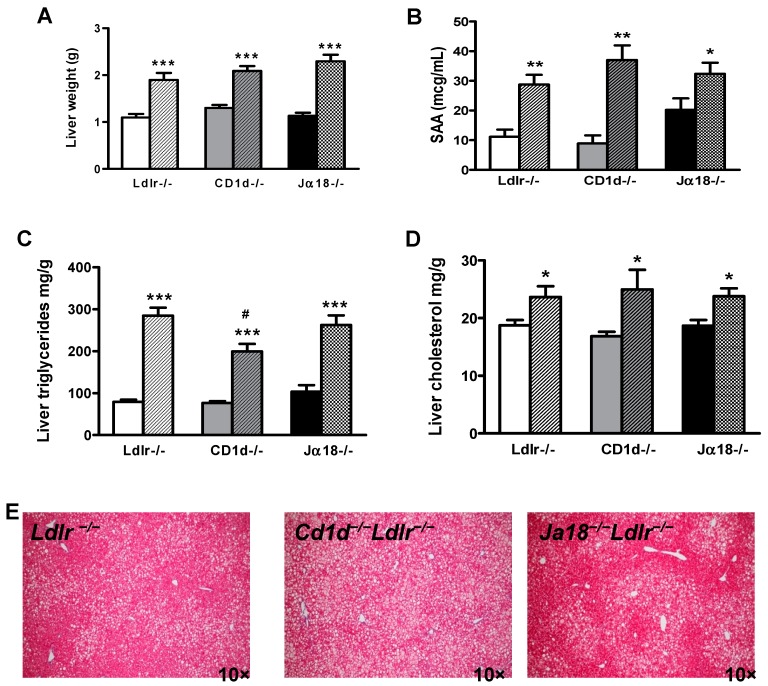

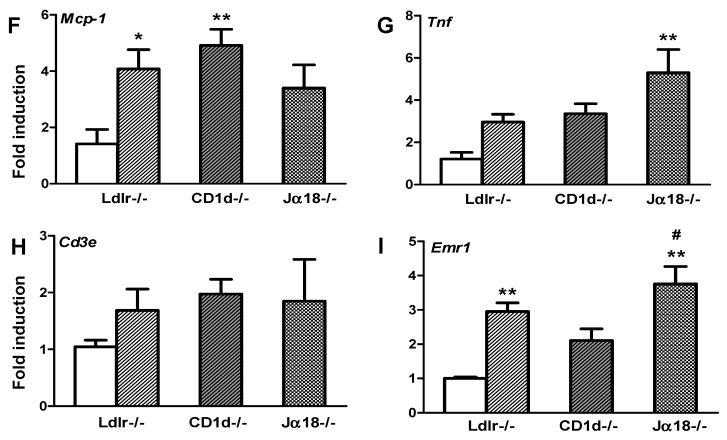
Effects of NKT cell deficiency in liver. (**A**) Liver weights, (**B**) plasma SAA levels, and hepatic triglyceride (**C**), and cholesterol (**D**) content. (**E**) Liver sections stained with Masson’s trichrome, 10× magnification. (**F**–**I**) Expression level of genes in whole liver tissue expressed as relative to lean chow-fed *Ldlr^−/−^* mice. *n* = 10 per group. * *p* < 0.05, ** *p* < 0.01, *** *p* < 0.001 vs. corresponding lean group, # *p* < 0.05 vs. HFHSC-fed *Ldlr^−/−^* mice. Solid bars—chow-fed animals; hatched bars—HFHSC-fed animals. SAA: serum amyloid A.

Changes in the liver lipid homeostasis are frequently seen with the feeding of a high fat diet. Since lipid sensing iNKT cells are highly enriched in the hepatic lymphocyte pool, we evaluated whether lack of iNKT cells can improve hepatic steatosis and inflammation. As with the adipose tissue, liver weights were higher in obese than in lean animals, but were not different between the three groups of mice (Figure 4A). Plasma circulating SAA levels, an inflammatory marker derived from the liver, were increased in all groups of obese mice (Figure 4B), with the levels being highest in the *Cd1d^−/−^Ldlr^−/−^* mice. Hepatic triglyceride content was increased in all mice on HFHSC diet relative to chow-fed animals, but was lower in the obese *Cd1d^−/−^Ldlr^−/−^* mice than the obese *Ldlr^−/−^* mice (Figure 4C). Hepatic cholesterol levels were higher in the HFHSC diets in each group compared to chow-fed controls (*p* < 0.05, Figure 4D). Evidence of hepatic steatosis was observed in all obese groups of mice (Figure 4E). Increased expression of the macrophage specific gene *Emr1* (encoding F4/80) with evidence of pro-inflammatory activation (*Tnfα*) was primarily observed in the obese *Ja18^−/−^Ldlr^−/−^* mice (*p* < 0.01, Figure 4F–I). 

### 2.5. Aortic Atherosclerosis Is Increased in Ja18^−/−^Ldlr^−/−^ Mice

**Figure 5:**
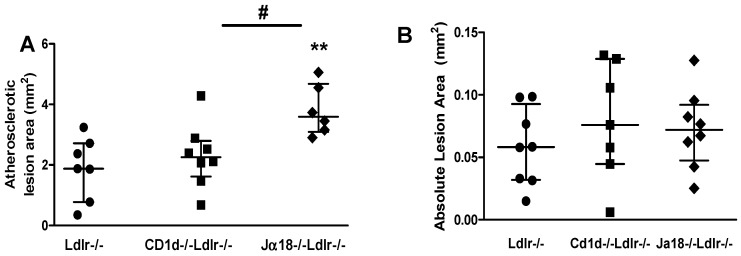

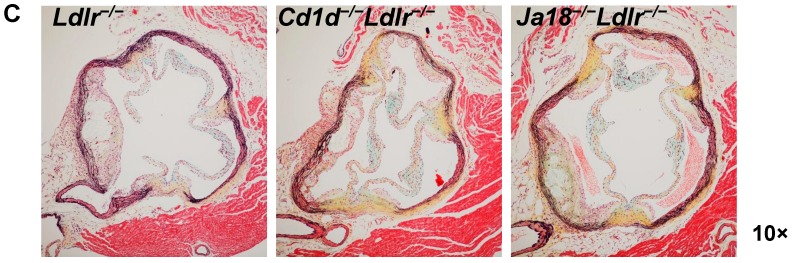
Aortic atherosclerosis is increased in obese *Ja18^−/−^Ldlr^−/−^* mice. (**A**) Quantitation of aortic intimal lesions; (**B**) Quantitation of aortic sinus lesions; (**C**) Photomicrographs of Movat’s pentachrome stained aortic sinus lesions. 10× magnification. *n* = 6–8 per group, ** *p* < 0.01 vs. HFHSC-fed *Ldlr^−/−^* mice, # *p* < 0.05 vs. HFHSC-fed *Cd1d^−/−^Ldlr^−/−^* mice.

Increased number or activation of iNKT cells has been shown to enhance atherosclerotic lesion development [17,30,31,32] and deficiency of NKT cells to decrease atherosclerosis [31,32,33,34,35]. To assess atherosclerosis in our diet-induced obesity models of NKT cell deficiency, we measured aortic atherosclerotic lesion area using the *en face* technique. Increased aortic atherosclerosis was observed in the obese *Ja18^−/−^Ldlr^−/−^* mice (*p* < 0.01, Figure 5A) lacking iNKT cells, but not in *Cd1d^−/−^Ldlr^−/−^* mice lacking both classes of NKT cells. However, no significant differences were seen at the aortic sinus (Figure 5B,C). 

## 3. Discussion

The *Ldlr*^−/−^ mouse develops many features of the metabolic syndrome when fed diets rich in saturated fat and refined carbohydrates. These effects are exacerbated by the addition of moderate amounts of dietary cholesterol [29]. We have recently shown that Vα14tg *Ldlr^−/−^* mice, which have an increased complement of iNKT cells throughout the development of obesity, are more obese and have increased inflammation, glucose intolerance, and atherosclerosis when fed this obesogenic diet [17]. The current study extends the analysis of the effects of NKT cells, again on the *Ldlr^−/−^* background, on obesity and atherosclerosis, in this case, examining the effect of NKT cell deficiency. Together, the studies represent the first comparisons of NKT cell abundance and deficiency, as they influence obesity in the context of hyperlipidemia in the *Ldlr^−/−^* background. Importantly, also they were performed in the same vivarium using the same obesogenic diet. In contrast to what we expected based on our previous study, in the current study, we find that the phenotype of HFHSC-fed *Ldlr^−/−^* mice lacking iNKT cells (*Ja18^−/−^Ldlr^−/−^* mouse) was, in many respects, surprisingly similar to that observed in the Vα14tg *Ldlr^−/−^* mice. Contrasted with control *Ldlr^−/−^* mice, as well as *Cd1d^−/−^Ldlr^−/−^* mice that lack the entire complement of NKT cells, the *Ja18^−/−^Ldlr^−/−^* mice that lack only iNKT cells exhibited increased weight gain, hyperlipidemia, adipose tissue inflammation, as well as increased aortic atherosclerosis.

The current consensus of existing studies is that iNKT cells decline markedly in adipose tissue in the setting of diet-induced obesity. The effect of the absence of iNKT on obesity and adipose tissue inflammation using *Ja18^−/−^* mice reported in the literature has been contradictory [28]. These diet-induced obesity studies have utilized a Western type diet or a high fat diet (40% or 60% calories) without added cholesterol in *Ldlr^+/+^* animals. In our studies, the genetic manipulations of NKT cells were in the *Ldlr^−/−^* background, enabling us to study metabolic effects of a hypercaloric diet, as well as to evaluate atherosclerosis in the same model. Our results on weight gain and adipose tissue inflammation with the *Ja18^−/−^Ldlr^−/−^* mice are consistent with several [11,16], but not all studies [19,24] using *Ja18^−/−^Ldlr^+/+^* mice.

The recent description of a sublineage of iNKT cells in adipose tissue as regulatory cells [20] may help explain our results with the *Ja18^−/−^Ldlr^−/−^* mice. This study by Lynch and colleagues determined that adipose tissue iNKT cells have a phenotype that is distinct from that of the iNKT cells of the liver and spleen in that adipose tissue iNKT cells express low levels of the characteristic transcription factor PLZF and high levels of IL-2 and IL-10, even at basal level and little IFNγ, upon stimulation. These NKT cells with regulatory properties, however, do not express the canonical Foxp3 found in T regulatory (Treg) cells [21]. The anti-inflammatory IL-2 and IL-10 cytokines promote the proliferation and function of Treg cells and the polarization of macrophages to the anti-inflammatory M2 subtype. Thus, in mice deficient in iNKT cells, the suppressive actions of iNKT cells in the adipose tissue are absent, and this could account for our observations. However, this does not fully account for the similarity in the phenotype of iNKT deficiency as reported here, and the phenotype of the *Ldlr^−/−^* animals that have an overabundance of iNKT cells. One possible explanation for this apparent paradox may be that the nature of the NKT cells in the adipose tissue of the Vα14 transgenic mice do not resemble the suppressive phenotype of iNKT cells seen in normal adipose tissue from wild type mice as described by Lynch and colleagues. The adipose tissue Vα14 iNKT cells may be more similar to the proinflammatory Th1-like iNKT cells found in the liver and spleen of wild type mice with the expression of IFNγ along with IL-4 [7]. So, a possible explanation for the paradox is that the increased inflammation in the adipose tissue of the transgenic mice is due to increased number of proinflammatory iNKT cells, while in the *Ja18^−/−^* mice, it is due to the absence of suppressive iNKT cells.

Tregs are an interesting partner for cross talk with iNKT cells. In visceral adipose tissue of lean aged male mice, Tregs constitute ~50% of all CD4+ T cells in the tissue [36]. Treg levels decrease in adipose tissue in genetic models of obesity and upon feeding a high fat diet [36,37]. Loss-of-function experiments using diphtheria toxin receptor expression, specifically in Tregs, resulted in increased adipose inflammation and decreased insulin signaling in adipocytes [36]. Conversely, increasing Tregs using IL-2-anti-IL-2 complexes resulted in increased insulin sensitivity [36]. Thus, the absence of iNKT cells could result in lower Treg numbers [20] that would increase inflammation. Treg cells are not homogeneous, and the spectrum of Tregs may differ from one tissue to another, as has been shown by Mathis and colleagues [1]. iNKT cells may interact with particular subsets of Tregs, allowing for complex metabolic interactions. An additional possibility is that there may be a reduction or skewing of the TCRα repertoire diversity in the knockout mouse strains, especially in the *Ja18^−/−^Ldlr^−/−^* mice [38], that may alter antigen recognition and activation.

Another unexpected result was that *CD1d^−/−^Ldlr^−/−^* mice are less obese, and exhibit overall less adipose tissue inflammation than is described in the other two NKT cell models we have studied, Vα14tg *Ldlr^−/−^* and *Ja18^−/−^Ldlr^−/−^* mice. CD1d is the antigen-presenting molecule for all NKT cells, iNKT and the more diverse type II NKT cells. In the absence of CD1d, both types of NKT cells are absent. Due to the requirement of CD1d for the maintenance of iNKT, one would have expected the phenotype of the adipose tissue in *Cd1d^−/−^Ldlr^−/−^* mice to be similar to that observed for the *Ja18^−/−^Ldlr^−/−^* mice. Three possibilities may account for these unexpected results. First is the possibility that type II NKT cells counteract the activity of iNKT cells, and are necessary for the suppressive activity of the iNKT cells in adipose tissue. Second, it is possible that CD1d, known to be expressed on epithelial cells including adipocytes, could, through signaling in these cells, counteract the suppressive action of iNKT cells. Third, is the possibility that the absence of CD1d in intestinal cells influences the intestinal microbiome, which in turn, modifies the obesity phenotype [39,40]. This could be mediated in part by the absence of CD1d-regulated secretion of anti-microbial peptides by Paneth cells [41].

We have studied these models in the *Ldlr* deficient background so that we are able to assess the effects of modulating NKT cells on atherosclerosis. Similar to the obesity phenotype, Vα14 transgenic animals [17] and *Ja18^−/−^* animals, but not *Cd1d^−/−^* animals exhibited increased aortic atherosclerosis. The transgenic animals displayed increased plasma cholesterol and lipoproteins, which could have played a role in the increment in atherosclerosis. However, perivascular adipose tissue has been shown to impact atherosclerosis [42,43,44]. So, the arguments raised in relation to NKT cells and adipose tissue inflammation could be relevant to periaortic adipose tissue modification of the underlying lesion development in the aorta.

In this and our previous paper, we have, for the first time, explored the role of different levels and subtypes of NKT cells on adipose tissue inflammation and atherosclerosis in *Ldlr^−/−^* mice housed in the same vivarium and fed the same obesogenic, high cholesterol diet. It is clear that the role of NKT cells in diet-induced obesity and atherosclerosis in male *Ldlr^−/−^* mice is quite complex, and requires much further exploration.

## 4. Materials and Methods

### 4.1. Animals and Diets

*Ja18^−/−^* mice obtained from Dr. Albert Bendelac (University of Chicago) and *CD1d^−/−^* mice were obtained from Dr. Chyung-Ru Wang (Northwestern University) were crossed with *Ldlr^−/−^* mice. All animals were in the C57BL/6J background. *Ldlr^−/−^* mice were used as controls. The absence of iNKT cells in the spleen and liver of *Ja18^−/−^Ldlr^−/−^* and *Cd1d^−/−^Ldlr^−/−^* mice was confirmed by the absence of CD3^+^αGalCer-tetramer^+^ (NIH Tetramer Core Facility) or CD3^+^ NK1.1^+^ cells by flow cytometry, as previously described [17]. Ten-week-old male mice were fed standard chow or a high fat, high sucrose diet with 0.15% cholesterol (HFHSC, Bioserv F4997, Frenchtown, NJ, USA) for 16 weeks (*n* = 10 per group). Mice were maintained in a temperature and light-controlled facility in cages with micro-isolator filter tops. Body weights were measured weekly. Food intake was recorded after 10 weeks of diet. At sacrifice, harvested tissues were snap-frozen in liquid nitrogen and stored at −70 °C, or were fixed with 10% neutral-buffered formalin and embedded in paraffin wax. All experimental procedures were undertaken with approval from the Institution Animal Care and Use Committee of the University of Washington (Seattle, WA, USA, 3104-01, 28 February 2016).

### 4.2. Analytical Procedures

Metabolic variables were measured in blood samples obtained from the retro-orbital sinus after a 5 h fast. Lipoproteins were separated from pooled plasma samples by fast-phase liquid chromatography (FPLC) using Superose 6 columns. Cholesterol and triglyceride levels in plasma and FPLC fractions were measured using colorimetric assay kits. Plasma insulin levels were measured using an ELISA kit (Millipore, Billerica, MA, USA). Tissue lipids were extracted using the Folch technique [45] and measured using colorimetric assay kits. Intra-peritoneal glucose and insulin tolerance tests were performed in 5 h fasted animals at weeks 13 and 14 of diet feeding, respectively, as previously described [29]. Body composition was measured using quantitative magnetic resonance (EchoMRI whole body composition analyzer, Echo Medical Systems, Houston, TX, USA). Plasma SAA levels were measured by ELISA, as previously described [46].

### 4.3. Real-Time Quantitative PCR

Total RNA was extracted from ~100 mg of whole adipose tissue and liver using a commercially available RNA extraction kit according to the manufacturer’s protocol (Agilent Technologies, Santa Clara, CA, USA). After spectroscopic quantification, 2 µg of RNA was reverse-transcribed, and the cDNA was analyzed by real-time quantitative PCR. Primers specific for individual genes were purchased from Applied Biosystems (Assay-on-Demand, Life Technologies, Carlsbad, CA, USA). GAPDH was used as the control housekeeping gene, levels of which did not change with diets. Relative amounts of the target gene were calculated using the ΔΔ*C*_t_ formula.

### 4.4. Histology, Immunohistochemistry, and Atherosclerosis Quantification

Formalin-fixed, paraffin-embedded adipose tissues were sectioned and stained using Movat’s pentachrome histochemical stain, using standard protocols. Macrophages in adipose tissue were detected using a rat monoclonal antibody (Mac2; titer 1:2500, Cedarlane Laboratories, Burlington, NC, USA). Liver sections were stained with Masson’s trichrome staining using standard protocols. The extent of atherosclerosis was measured in pinned aortas using the *en face* technique, and aortic sinus lesion analysis was performed as previously described [29].

### 4.5. Statistics

Data were analyzed using the GraphPad Prism 5 program (GraphPad Software Inc., La Jolla, CA, USA) and are represented as means and standard errors. Student’s *t*-test was used to detect differences within groups when applicable. One-way analysis of variance (ANOVA) was used to compare differences among all groups, and Bonferroni post hoc testing was used to detect differences among mean values of the groups. A *p* value < 0.05 was considered as statistically significant.

## Abbreviations

FPLC	fast performance liquid chromatography
HFHSC	high fat, high sucrose, cholesterol
iNKT	invariant Natural Killer T
Ldlr	Low density lipoprotein receptor
SAA	serum amyloid A
TCR	T cell receptor
Treg	T regulatory
